# Supplementary material for: Probing the physiological role of the plastid outer-envelope membrane using the oemiR plasmid collection
Source: G3 (Bethesda). 2023 Aug 12;13(10):jkad187. doi: 10.1093/g3journal/jkad187 (PMC10542568; doi:10.1093/g3journal/jkad187)
Supplement: jkad187_Supplementary_Data [file jkad187_supplementary_data.zip › Supplementary_Material_Legends_G3-2023-404435.docx]

**Supplementary data**

**Supplementary Data S1:** Redundancy predictions and information on the oemiR plasmid collection

**Supplementary Data S2**: Vector maps of the oemiR plasmid collection.

**Supplementary Data S3:** Proteome analysis of *amiR-toc75* mutants.
